# Detection and Molecular Characterization of Zoonotic Poxviruses Circulating in the Amazon Region of Colombia, 2014

**DOI:** 10.3201/eid2304.161041

**Published:** 2017-04

**Authors:** Jose A. Usme-Ciro, Andrea Paredes, Diana M. Walteros, Erica Natalia Tolosa-Pérez, Katherine Laiton-Donato, Maria del Carmen Pinzón, Brett W. Petersen, Nadia F. Gallardo-Romero, Yu Li, Kimberly Wilkins, Whitni Davidson, Jinxin Gao, Nishi Patel, Yoshinori Nakazawa, Mary G. Reynolds, P. S. Satheshkumar, Ginny L. Emerson, Andrés Páez-Martínez

**Affiliations:** Instituto Nacional de Salud, Bogotá, Colombia (J.A. Usme-Ciro, A. Paredes, D.M. Walteros, E.N. Tolosa-Pérez, K. Laiton-Donato, A. Páez-Martínez); Universidad Cooperativa de Colombia, Santa Marta, Colombia (J.A. Usme-Ciro);; Laboratorio Departamental de Salud de Caquetá, Caquetá, Colombia (M. del Carmen Pinzón);; Centers for Disease Control and Prevention, Atlanta, USA (B.W. Petersen, N.F. Gallardo-Romero, Y. Li, K. Wilkins, W. Davidson, J. Gao, N. Patel, Y. Nakazawa, M.G. Reynolds, P.S. Satheshkumar, G.L. Emerson);; Universidad de La Salle, Bogotá (A. Páez-Martínez)

**Keywords:** poxvirus, vaccinia virus, pseudocowpox virus, zoonoses, dairy farmworkers, cattle, Colombia, Amazon, outbreak, viruses

## Abstract

During 2014, cutaneous lesions were reported in dairy cattle and farmworkers in the Amazon Region of western Colombia. Samples from 6 patients were analyzed by serologic and PCR testing, and results demonstrated the presence of vaccinia virus and pseudocowpox virus. These findings highlight the need for increased poxvirus surveillance in Colombia.

The *Poxviridae* family comprises large double-stranded DNA viruses that infect a wide range of invertebrate and vertebrate animals, including humans (*1*). Many poxviruses, particularly those belonging to the genus *Orthopoxvirus* (e.g., vaccinia virus [VACV]) and *Parapoxvirus* (e.g., pseudocowpox virus [PCPV]), are considered zoonotic viruses because their infections usually arise from human contact with infected domestic or sylvatic animal species (*2*).

Orthopoxviruses (OPXVs) gained considerable attention in the past because variola (smallpox) virus fatally infected millions of persons worldwide for centuries. However, in 1980, smallpox became the first infectious disease to be eradicated, resulting from vaccination campaigns involving several VACV strains (*3*). VACV has been detected in recurrent zoonotic outbreaks in Brazil, where it has been categorized into 2 well-defined genetic lineages (*4*). VACV has also emerged as a zoonotic virus in India (*5*). In 2011, VACV infections were found in cattle herds during active surveillance in Argentina (*6*). In Colombia, the most recently reported VACV outbreak in 1965–1966 caused disease in ≈8,570 cows and 150 humans and was associated with intensified smallpox eradication campaigns that extended until 1972 (*3*,*7*).

Parapoxviruses are emerging zoonotic pathogens that can cause infections in humans through direct contact with infected animals, especially domestic and wild ruminants (*1*). Among these viruses, bovine papular stomatitis virus, orf virus, and PCPV are known to cause cutaneous lesions (*2*). Although a parapoxvirus outbreak in imported goats was reported in Colombia in 1983 (*8*), only Brazil and the United States have formal evidence of endemic and zoonotic transmission of these viruses (*9*,*10*). We describe the clinical features, serologic and molecular diagnosis, and phylogenetic analysis of poxviruses circulating along the Amazon in western Colombia.

## The Study

**Figure 1:**
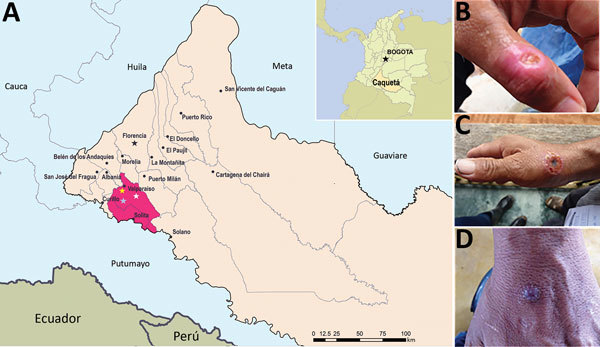
Location of 6 patients with poxvirus infections and photographs of lesions from 3 patients, Colombia, 2014. A) The municipalities of Valparaíso (residence of patients 1–5) and Solita (residence of patient 6) are 36 km apart in the southwestern region of the department of Caquetá. Yellow, white, and blue stars denote locations of patients 2, 3, and 4, respectively. Inset shows location of Caquetá in Colombia. Map source: Departamento Administrativo Nacional de Estadística (http://geoportal.dane.gov.co/). B–D) Exanthematous lesions on the hands of dairy farm workers depicting the varying sizes of lesions and typical evolution of ulcers. Patient 2 (B) tested positive for vaccinia virus, patient 3 (C) had inconclusive results, and patient 4 (D) was positive for parapoxvirus.

During an active outbreak investigation in February–July 2014, serum and exanthematous lesion samples were collected from 6 patients in the bordering municipalities of Valparaíso (patients 1–5) and Solita (patient 6) in the department of Caquetá (Figure 1; Table) in the Amazon Region of Colombia. Archived serum samples collected in December 2012–April 2013 from 11 patients with exanthematous lesions (from several farms) in the municipality of Valparaíso who consulted the local hospital were also analyzed (Table). In all patients, the incubation period ranged from 4–7 days, after which nodules on the hands or forearms appeared, along with fever, lymphadenopathy, and localized pain. Lesions increased in size and progressed from erythematous macules to papules, vesicles, and pustules that subsequently exhibited bacterial infection by the fourth week of symptom onset. By the fifth and sixth week, lesions progressed to scabs (Figure 1). Lesions appeared mainly in hands with preexisting cuts, abrasions, or other skin barrier defects. Direct contact with lesions from the udders of cattle during milking was reported by all but 1 patient. Patient 5 was a 24-year-old woman who reported no recent contact with infected animals, but her husband was a milker who had exanthematous lesions on his hands 2 weeks before she displayed symptoms. 

**Table:** Orthopoxvirus IgG and IgM antibodies and poxvirus genome detection in lesion and serum samples*

Patient no.	Identification no.	Sex	Age, y	Vaccination status	Municipality	ELISA			Real-time PCR (C_t_)	
						IgG†	IgM‡		VACV	Parapoxvirus
1	2014030600020	F	13	Unvaccinated	Valparaíso	Pos (0.176)	Pos (0.408)		Pos (21.8)	Neg
2	2014030600021	F	15	Unvaccinated	Valparaíso	Pos (0.393)	Pos (0.450)		Pos (27.8)	Neg
3	2014030600022	M	46	UNK	Valparaíso	Pos (0.327)	Pos (1.102)		Inc	Inc
4	2014030600023	M	34	Unvaccinated	Valparaíso	Pos (0.166)	Neg		Neg	Pos (36.8)
5	2014042400031 (POX0001)	F	24	Unvaccinated	Valparaíso	Neg	Neg		Pos (21.6)	Neg
6	2014062800100 (POX0002)	M	UNK	UNK	Solita	Neg	Neg		Neg	Pos (35.9)
7	2013050600018	M	26	Unvaccinated	Valparaíso	Pos (0.254)	Pos (0.898)		Neg	Neg
8	2013050600026	M	52	UNK	Valparaíso	Pos (0.213)	Neg		Neg	Neg
9	2013050600019	M	17	Unvaccinated	Valparaíso	Pos (0.261)	Eqi (0.094)		Neg	Neg
10	2013050600020	M	20	Unvaccinated	Valparaíso	Pos (0.384)	Neg		Neg	Neg
11	2013050600021	M	47	UNK	Valparaíso	Pos (0.383)	Pos (0.632)		Neg	Neg
12	2013050600022	M	18	Unvaccinated	Valparaíso	Pos (0.450)	Pos (0.202)		Neg	Neg
13	2013050600027	M	27	Unvaccinated	Valparaíso	Pos (0.224)	Pos (0.243)		Neg	Neg
14	2013050600023	M	24	Unvaccinated	Valparaíso	Pos (0.482)	Neg		Neg	Neg
15	2013050600024	F	12	Unvaccinated	Valparaíso	Pos (0.272)	Pos (0.128)		Neg	Neg
16	2013050600028	M	13	Unvaccinated	Valparaíso	Pos (0.451)	Pos (0.427)		Neg	Neg
17	2013050600025	M	23	Unvaccinated	Valparaíso	Pos (1.651)	Eqi (0.029)		Neg	Neg

*C_t_, cycle threshold; eqi, equivocal; inc, inconclusive; neg, negative; pos, positive; UNK, unknown. †OPXV IgG ELISA titers correspond to a 1:100 dilution of the serum sample.  ‡OPXV IgM ELISA titers correspond to a 1:50 dilution of the serum sample.

All serum samples were tested by ELISA for OPXV IgG and IgM as described (*11*) (Technical Appendix). An ELISA for detecting parapoxvirus-specific antibodies was not available. Fifteen serum samples were positive for OPXV IgG (Table); 9 were also positive for OPXV IgM, which can persist for up to 6 months after primary infection or vaccination (*11*). Reliable vaccination data was not available for these patients; however, on the basis of age, only patients 3, 8, and 11 could have been vaccinated. These 3 patients exhibited detectable OPXV IgG levels, and patients 3 and 11 were also positive for OPXV IgM. These results suggest that these patients had not been vaccinated and that their lesions were caused by recent OPXV exposure. Patients 4, 10, and 14 exhibited detectable OPXV IgG without detectable OPXV IgM. These results suggest the patients had previous OPXV infections, given that an active parapoxvirus infection was demonstrated in patient 4 and vaccination could be ruled out for patients 10 (a 20-year-old) and 14 (a 24-year-old) based on their age.

**Figure 2:**
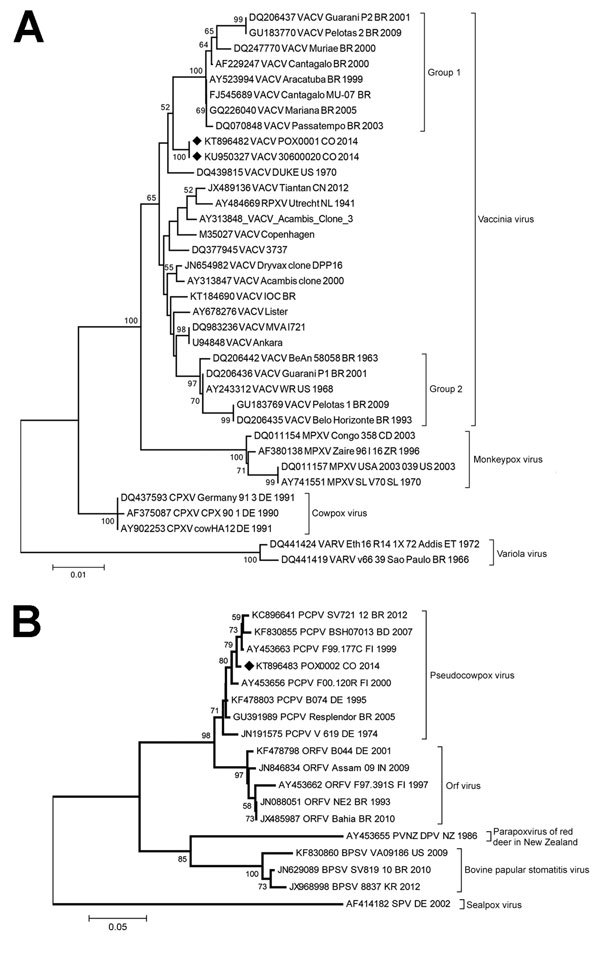
Phylogenetic characterization of orthopoxvirus gene *A56R* and parapoxvirus gene *p37K* of viruses obtained from patient lesion samples from an outbreak in Colombia, 2014. Trees were inferred by the neighbor-joining method. A) Nucleotide sequences of the *A56R* gene (829 bp) of reference orthopoxvirus strains were aligned and used for phylogenetic inference. The evolutionary distances were computed by using the T92+G model (shape: 0.69). Vaccinia virus (VACV) groups 1 and 2 are labeled with brackets. B) Nucleotide sequences of the partial (445 bp) *p37K* gene of reference parapoxvirus strains were aligned and used for phylogenetic inference. The evolutionary distances were computed by using the T92+G model (shape: 0.39). The percentage of replicate trees in which the associated taxa clustered together in the bootstrap test (1,000 replicates) are shown at the nodes. Diamonds indicate poxvirus isolates from Colombia. GenBank accession numbers and further information on the sequences included in the analyses are provided in Technical Appendix Tables 1, 2. Scale bars indicate nucleotide substitutions per site. BPSV, bovine papular stomatitis virus; CPXV, cowpox virus; RPXV, rabbitpox virus; MPXV, monkeypox virus; ORFV, orf virus; PCPXV, pseudocowpox virus; PVNZ, parapoxvirus of red deer in New Zealand; SPV, sealpox virus; VARV, variola virus.

DNA was extracted from lesion and serum samples by using the PureLink viral RNA/DNA extraction kit (Invitrogen Inc., Carlsbad, CA, USA) according to the manufacturer’s instructions and amplified by using PCR with generic and specific poxvirus primers as previously described (Technical Appendix) (*12*). OPXV DNA was detected in lesion samples from patients 1, 2, and 5 (Table). For further genetic characterization, the *A56R* (hemagglutinin, 1,134 bp) gene from patients 2 and 5 were amplified by PCR, sequenced (Technical Appendix), and compared with reference orthopoxvirus *A56R* sequences (Technical Appendix Table 1). Our phylogenetic analysis confirmed VACV to be the etiologic agent of infection in these patients (Figure 2, panel A). The OPXV isolates detected in our samples were related to group 1 (Passatempo, Aracatuba, Muriae, and other strains from Brazil); however, bootstrap score and branch positioning suggest the strains from Brazil and Colombia diverged long ago or independently arose.

For parapoxvirus detection and characterization, we designed generic and degenerate primers to amplify a partial sequence of the *p37K* (envelope protein B2L, 489 bp) gene by PCR (Technical Appendix). Parapoxvirus DNA was detected in lesion samples from patients 4 and 6 (Table). Nucleotide sequencing and phylogenetic analysis of the sample from patient 6 showed a close relationship with previously reported PCPV strains circulating in Brazil and worldwide (Technical Appendix Table 2; Figure 2, panel B).

## Conclusions

We found OPXV IgG and IgM in serum samples collected from patients with exanthematous lesions and from those who had such lesions in the past. Molecular methods allowed for the detection and characterization of VACV (3 patients) and PCPV (2 patients). Lesions caused by poxviruses affected the hands and forearms and disappeared within weeks without treatment. However, by limiting the patients’ daily activities, the infections had substantial negative impacts on the economies of dairy farmworkers and their local communities.

The disease burden of poxvirus infections in Colombia has not been estimated. However, anecdotal communications and data suggest it might be a serious and increasing health problem because farmworkers and healthcare personnel are not trained to recognize the disease and prevent subsequent transmission. The rapid spread of poxviruses could be facilitated by farmworkers performing daily milking activities at several farms and trading cattle. Also, factors such as human-to-human and fomite transmission, a growing susceptible population, and an unknown animal reservoir might increase the potential risk for infection at the community level. The serious consequences that poxvirus infections could have on immunocompromised persons (*13*), the increased risk for transmission related to the anatomic site of the lesions (*14*), the potential for long-lasting sequelae, and the possibility of the virus evolving into a more virulent strain (*15*) could pose a great threat to individual persons, populations, and public health systems in the near future.

Studies focused on determining the prevalence of poxvirus infections, risk factors for disease, and the geographic distribution of poxvirus strains are needed to understand the disease burden and guide effective prevention and control measures and educational outreach. Analysis and characterization of VACV and PCPV complete genomes could provide clues to explain their emergence and recent evolutionary histories, and research aimed at identifying the domestic animal hosts and wildlife reservoirs of poxviruses could further our understanding of their natural transmission cycles.

Technical Appendix Description of methods and information on the *A56R* and *p37K* reference genes used in phylogenetic analyses.
