# Supplementary material for: Detection and Molecular Characterization of Zoonotic Poxviruses Circulating in the Amazon Region of Colombia, 2014
Source: Emerg Infect Dis. 2017 Apr;23(4):649–53. doi: 10.3201/eid2304.161041 (PMC5367405; doi:10.3201/eid2304.161041)
Supplement: Technical Appendix — Description of methods and information on the A56R and p37K reference genes used in phylogenetic analyses. [file 16-1041-Techapp-s1.pdf]

Article DOI: http://dx.doi.org/10.3201/eid2304.161041

Detection and Molecular Characterization of Zoonotic Poxviruses Circulating in the Amazon Region of Colombia, 2014

Technical Appendix

Laboratory Methods

Serologic Tests

Orthopoxvirus (OPXV) IgM and IgG in patient serum samples were detected by ELISA as described previously (*1*). Known positive and negative serum samples were used as assay controls, and cutoff values were assigned by adding 3 standard deviations to the mean of the control serum sample readings. The cutoff value was subtracted from the average optical density (OD) for each sample dilution to determine the value for analysis. For OPXV IgM assays, values of samples at the 1:50 dilution were classified as positive (>0.1), negative (<0), or inconclusive (0–0.1). Values >0 at 1:100 dilution were considered positive in OPXV IgG assays.

Viral DNA Extraction

Exanthematous lesion samples were mixed with 300 μL of lysis buffer (containing 1 M Tris-HCl, 20% SDS, 0.5 M EDTA (pH 8.0), 20 mg/mL proteinase K, and 10 U/μL RNase inhibitor) and incubated at 60°C for 16 h. DNA was extracted from lysed lesion and serum samples by using the PureLink viral RNA/DNA extraction kit (Invitrogen Inc., Carlsbad, CA, USA), according to the manufacturer's instructions. DNA was eluted in 30 μL of nuclease-free water and stored at −20°C.

PCR Amplification

The complete *A56R* (hemagglutinin, 1,134 bp) gene was PCR amplified and sequenced with primers A56R_F (5′-AACACAGTACACGAATGATTTTCT-3′) and A56R_R (5′-TTGGCTATGGCTGTCTTTCC-3′), designed by using the PrimerSelect module of the LaserGene version 7.2.1 (DNASTAR Inc., Madison, WI, USA). PCR primers Parapox_F (5′-TCCACGCTACCSACGCCRAAG-3′) and Parapox_R (5′-

TCCGTGTCCAMCATCAARAACC-3′) used for amplification and detection of a 489-bp fragment of the *p37K* gene encoding the viral envelope antigen of members of the *Parapoxvirus* genus (pseudocowpox, orf virus, and bovine popular stomatitis virus) were designed as described above. To allow for genetic variability among the parapoxviruses, some degenerate sites were included in the primer design and indicated in accordance with the nucleotide ambiguity code of the International Union of Pure and Applied Chemistry (http://www.chick.manchester.ac.uk/SiteSeer/IUPAC_codes.html). The amplification reaction mixes for *A56R* and *p37K* contained 0.4 mM of each dNTP, 2 mM $MgCl_2$, 10 pmol of each primer, 0.25 U *Taq* DNA polymerase (Life Technologies Corp., Carlsbad, CA, USA), and 5 µL of the previously extracted DNA in a final volume of 25 µL. Thermal cycling parameters for *A56R* and *p37K* were 94°C for 5 min, 40 cycles of amplification (94°C for 30 sec, 55°C for 30 sec, and 72°C for 1 min), and a final extension at 72°C for 5 min. PCR results were validated by real-time PCR assays for detection of parapoxvirus and vaccinia virus, as previously described (*2*).

Nucleotide Sequencing

Each amplicon sequence was prepared by using the previously stated forward and reverse oligonucleotides and BigDye Terminator Cycle Sequencing kit v.3.1 (Applied Biosystems, Foster City, CA, USA) according to the manufacturer's instructions. Reactions were purified with the BigDye XTerminator purification kit (Applied Biosystems) and sequenced on the 3130 Genetic Analyzer (Applied Biosystems). Reads were assembled through the SeqMan module of LaserGene version 7.2.1 (DNASTAR Inc.).

Phylogenetic Analysis

Sets of sequences were obtained for the *A56R* gene of orthopoxviruses and for the *p37K* gene of parapoxviruses from cases identified in Colombia and worldwide and aligned by using ClustalX2.1 software (*3*). Each matrix of aligned sequences was used to calculate the best nucleotide substitution model through the Bayesian information criterion implemented in jModelTest 2 (*4*,*5*). Estimates of genetic distances were obtained from Mega 6.06 (*6*). Phylogenetic relationships were determined by the neighbor-joining method implemented in Mega 6.06 using the previously estimated models of nucleotide substitution and 1,000 bootstrap replicates.

References

1. Karem KL, Reynolds M, Braden Z, Lou G, Bernard N, Patton J, et al. Characterization of acute-phase humoral immunity to monkeypox: use of immunoglobulin M enzyme-linked immunosorbent assay for detection of monkeypox infection during the 2003 North American outbreak. Clin Diagn Lab Immunol. 2005;12:867–72. PubMed

2. Zhao H, Wilkins K, Damon IK, Li Y. Specific qPCR assays for the detection of orf virus, pseudocowpox virus and bovine papular stomatitis virus. J Virol Methods. 2013;194:229–34. PubMed http://dx.doi.org/10.1016/j.jviromet.2013.08.027

3. Larkin MA, Blackshields G, Brown NP, Chenna R, McGettigan PA, McWilliam H, et al. Clustal W and clustal X version 2.0. Bioinformatics. 2007;23:2947–8. PubMed http://dx.doi.org/10.1093/bioinformatics/btm404

4. Darriba D, Taboada GL, Doallo R, Posada D. jModelTest 2: more models, new heuristics and parallel computing. Nat Methods. 2012;9:772. PubMed http://dx.doi.org/10.1038/nmeth.2109

5. Guindon S, Gascuel O. A simple, fast, and accurate algorithm to estimate large phylogenies by maximum likelihood. Syst Biol. 2003;52:696–704. PubMed http://dx.doi.org/10.1080/10635150390235520

6. Tamura K, Stecher G, Peterson D, Filipski A, Kumar S. MEGA6: Molecular Evolutionary Genetics Analysis version 6.0. Mol Biol Evol. 2013;30:2725–9. PubMed http://dx.doi.org/10.1093/molbev/mst197

Technical Appendix Table 1. List of orthopoxvirus *A56R* gene sequences used in the phylogenetic analysis*

GenBank accession no.	Strain	Species	Year	Country
KT896482	POX0001	VACV	2014	Colombia
KU950327	30600020	VACV	2014	Colombia
DQ070848	Passatempo	VACV	2003	Brazil
DQ206435	Belo_Horizonte	VACV	1993	Brazil
GU183770	Pelotas_2	VACV	2009	Brazil
DQ247770	Muriae	VACV	2000	Brazil
DQ206437	Guarani_P2	VACV	2001	Brazil
GU183769	Pelotas_1	VACV	2009	Brazil
DQ206442	BeAn_58058	VACV	1963	Brazil
DQ206436	Guarani_P1	VACV	2001	Brazil
AY523994	Araçatuba	VACV	1999	Brazil
AF229247	Cantagalo	VACV	2000	Brazil
FJ545689	Cantagalo MU-07	VACV	–	Brazil
GQ226040	Mariana	VACV	2005	Brazil
JX489136	Tiantan	VACV	2012	China
KT184690	IOC	VACV	–	Brazil
AY313847†	Acambis_ 2000	VACV	–	–
JN654982†	Dryvax_DPP16	VACV	–	–
AY313848†	Acambis_ 3	VACV	–	–
DQ377945†	3737	VACV	–	–
AY678276†	Lister	VACV	–	–
U94848†	Ankara	VACV	–	–

GenBank accession no.	Strain	Species	Year	Country
M35027†	Copenhagen	VACV	–	–
AY243312	WR	VACV	1968	United States
DQ983236†	MVA_I721	VACV	–	–
DQ439815	DUKE	VACV	1970	United States
AF380138	Zaire_96_I_16	MPXV	1996	Zaire
DQ011157	USA_2003_039	MPXV	2003	United States
AY741551	SL_V70	MPXV	1970	Sierra Leone
DQ011154	Congo_358	MPXV	2003	Congo
DQ437593	Germany_91_3	CPXV	1991	Germany
AF375087	CPX_90_1	CPXV	1990	Germany
AY902253	cowHA12	CPXV	1991	Germany
DQ441424	Eth16_R14_1X_72	VARV	1972	Ethiopia
DQ441419	v66_39_Sao_Paulo	VARV	1966	Brazil
AY484669	Utrecht	RPXV	1941	Netherlands

*CPXV, cowpox virus; MPXV, monkeypox virus; RPXV, rabbitpox virus; VACV, vaccinia virus; VARV, variola virus; –, unknown.
†Widely used vaccine strains for which year of isolation and location are not available.

Technical Appendix Table 2. List of parapoxvirus partial *p37K* gene sequences used in the phylogenetic analysis*

GenBank accession no.	Strain	Species	Year	Country
KT896483	POX0002	PCPV	2014	Colombia
AY453656	F00.120R	PCPV	2000	Finland
AY453663	F99.177C	PCPV	1999	Finland
JN191575	V_619	PCPV	1974	Germany
GU391989	Resplendor	PCPV	2005	Brazil
KC896641	SV721_12	PCPV	2012	Brazil
KF478803	B074	PCPV	1995	Germany
KF830855	BSH07013	PCPV	2007	Bangladesh
AY453662	F97.391S	ORFV	1997	Finland
JN088051	NE2	ORFV	1993	Brazil
JN846834	Assam_09	ORFV	2009	India
JX485987	Bahia	ORFV	2010	Brazil
KF478798	B044	ORFV	2001	Germany
JN629089	SV819_10	BPSV	2010	Brazil
JX968998	8837	BPSV	2012	South Korea
KF830860	VA09186	BPSV	2009	United States
AY453655	DPV	PVNZ	1986	New Zealand
AF414182	SPV_DE_2002	SPV	2002	Germany

*PCPV, pseudocowpox virus; ORFV, orf virus; BPSV, bovine papular stomatitis virus; PVNZ, parapoxvirus of red deer in New Zealand; SPV, sealpox virus.
